# Methylation of HIN-1, RASSF1A, RIL and CDH13 in breast cancer is associated with clinical characteristics, but only RASSF1A methylation is associated with outcome

**DOI:** 10.1186/1471-2407-12-243

**Published:** 2012-06-13

**Authors:** Jia Xu, Priya B Shetty, Weiwei Feng, Carol Chenault, Robert C Bast, Susan G Hilsenbeck, Yinhua Yu

**Affiliations:** 1 Department of Experimental Therapeutics, The University of Texas MD Anderson Cancer Center, 1515 Holcombe Blvd, Houston, TX, 77030, USA; 2 Dan L Duncan Cancer Center, Baylor College of Medicine, 1 Baylor Plaza, Houston, TX, 77030, USA; 3 Department of Leukemia, The University of Texas MD Anderson Cancer Center, 1515 Holcombe Blvd, Houston, TX, 77030, USA; 4 Lester and Sue Smith Breast Center, Baylor College of Medicine, 1 Baylor Plaza, Houston, TX, 77030, USA; 5 Department of Epidemiology and Biostatistics, Case Western Reserve University School of Medicine, 10900 Euclid Avenue, Cleveland, OH, 44106, USA; 6 Graduate School of Biomedical Science, The University of Texas, Houston, TX, 77030, USA; 7 Obstetrics and Gynecology Hospital of Fudan University, Shanghai, 200011, China; 8 Fels Institute for Cancer and Molecular Biology, Temple University, 3307 North Broad Street, Philadelphia, PA, 19140, USA

## Abstract

**Background:**

Aberrant promoter CpG island hypermethylation is associated with transcriptional silencing. Tumor suppressor genes are the key targets of hypermethylation in breast cancer and therefore may lead to malignancy by deregulation of cell growth and division. Our previous pilot study with pairs of malignant and normal breast tissues identified correlated methylation of two pairs of genes - HIN-1/RASSFIA and RIL/CDH13 - with expression of estrogen receptors (ER), progesterone receptors (PR), and HER2 (HER2). To determine the impact of methylation on clinical outcome, we have conducted a larger study with breast cancers for which time to first recurrence and overall survival are known.

**Methods:**

Tumors from 193 patients with early stage breast cancer who received no adjuvant systemic therapy were used to analyze methylation levels of RIL, HIN-1, RASSF1A and CDH13 genes for associations with known predictive and prognostic factors and for impact on time to first recurrence and overall survival.

**Results:**

In this study, we found that ER was associated with RASSF1A methylation (*p* < 0.001) and HIN-1 methylation (*p* = 0.002). PR was associated with RIL methylation (*p* = 0.012), HIN-1 (*p* = 0.002), and RASSF1A methylation (*p* = 0.019). Tumor size was associated with RIL and CDH13 methylation (both *p* = 0.002), and S-phase was associated with RIL methylation (*p* = 0.036). Only RASSF1A was associated with worse time to first recurrence (*p* = 0.045) and worse overall survival (*p* = 0.016) after adjusting for age, tumor size, S-phase, estrogen receptor and progesterone receptor.

**Conclusions:**

Methylation of HIN-1, RASSF1A, RIL and CDH13 in breast cancers was associated with clinical characteristics, but only RASSF1A methylation was associated with time to first recurrence and overall survival. Our data suggest that RASSF1A methylation could be a potential prognostic biomarker.

## Background

Breast cancer is the leading cause of cancer-related death in women worldwide, but the exact etiology of breast cancer remains elusive. DNA methylation has attracted intensive investigation in recent years and it is thought to play an important role in the regulation of genes related to cancer [1]. Several tumor suppressor genes are silenced by promoter hypermethylation in breast cancers including HIN-1 [2], RASSF1A [3], RIL [4], and CDH13 [5], among others. Aberrant DNA methylation of particular genes has been correlated with the clinical and pathologic characteristics of breast cancers and with clinical outcomes [6].

It is widely recognized that subtypes identified through gene expression profiling are associated with distinct clinical outcomes and targets for treatment [7-10]. DNA methylation markers have been applied as an alternative approach to molecular profiling of breast cancer [5,6,11]. CDH13 has been reported to be highly methylated in HER2/neu-positive breast cancers, which comprise 20% to 30% of invasive breast carcinomas. Methylated CDH13 is associated with increased metastatic potential and lower tamoxifen sensitivity [11]. Higher methylation of HIN-1 has been found in bone, brain, and lung metastases compared to the associated primary breast carcinomas [12]. RASSF1A promoter methylation provides important prognostic information in early stage breast cancer patients [13]. All of these results suggest that DNA methylation profiling correlates with clinical status in breast cancer and could help predict response to hormonal and non-hormonal breast cancer therapy [5]. However, a persistent panel of DNA methylation markers which are correlated with clinic characteristics and outcomes in breast cancers has not been described in detail yet.

To investigate the associations between gene methylation profiles and hormone receptor status in patient samples, our previous study measured methylation of 12 known tumor-suppressor genes in 90 pairs of breast cancers and normal tissues, and we found that five genes proved useful in defining a methylation profile in breast cancer cells. We also found that two panels of methylation profiles (HIN-1/RASSF1A, and RIL/CDH13) correlated, either positively or negatively, with estrogen receptors (ER), progesterone receptors (PR) or Her-2-neu status [6]. This observation, while interesting, is of limited utility due to the small sample size and lack of outcome data. To confirm our observation and to determine whether methylation profiles of these particular genes have prognostic significance, we analyzed 193 well annotated breast cancers.

## Methods

### Tissue samples

For this study, we used breast cancer tissue samples and associated clinicopathologic data from the tissue banks at the Lester and Sue Smith Breast Center at Baylor College of Medicine. All tissue samples were obtained after receiving informed consent according to institutional rules. No patient was recruited specifically for this study which was approved by Institutional Review Boards of The University of Texas M.D. Anderson Cancer Center and of Baylor College of Medicine.

Tumor samples from 193 patients were obtained from the excess tissue collected during medically necessary procedures to excise primary breast cancers between 1973 and 1999. The tissue was flash-frozen and pulverized for steroid receptor assessment by ligand binding assay in one of two central laboratories. The remainder was kept for future research. This tissue resource was aliquoted and stored at −80 °C in cryovials. In June 2001, the area where these samples were stored was flooded, and samples thawed and were then refrozen 48 hours later. Although proteins and mRNA were degraded, moderate length DNA remained intact, and is useful for a variety of DNA based assays. The tissue banks which contributed tumor samples toward this study have been continuously approved since inception by relevant institutional review boards. None of the patients who provided the tumor samples chosen for this study was given adjuvant therapy.

### Prognostic factors

ER levels were measured by the dextran-coated charcoal method, as previously described [14]. From 1970 to 1984, ^3^ H] estradiol was used as the labeled ligand. During the same period, PR levels were measured by sucrose density gradient [15]. In 1985, the standard multipoint dextran-coated charcoal assay was modified to incorporate ^125^I] estradiol and ^3^ H] R5020 in a single assay, allowing the simultaneous determination of both ER and PR. Samples containing at least 3 fmol/mg protein were considered ER positive, and those containing at least 5 fmol/mg protein were considered PR positive, based on prior clinical studies [16-18].

DNA ploidy and S-phase fraction were evaluated by flow cytometry and the histograms were analyzed by Modfit (Verify Software House, Topsham, ME) using singlecut debris stripping [18]. Cutpoints were determined by calibrating S-phase fraction with clinical outcome in a group of more than 28,800 patients with breast cancer (low, <6%; intermediate, 6%–10%; high > 10%) [19].

### DNA extraction and sodium bisulfite treatment

In brief, genomic DNA was extracted from patient samples, breast cancer cell lines, and normal breast epithelial cells using the Dneasy tissue kit (Qiagen, Valencia, CA, USA). Bisulfite treatment of 1–2 μg of genomic DNA was performed, as previously described [20].

### Genes studied

Four tumor-suppressor genes, RASSF1A, SCGB3A1 (HIN-1), PDLIM4 (RIL) and CDH13 were selected for this study. Pyrosequencing methylation analysis was used to detect methylation of all 4 genes. Pyrosequencing primers were designed using Assay Design software 1.0 (Biotage, Westborough, MA, USA). For each gene, we selected the CpG island region flanking the transcription start site at the 5'UTR. Two to six CpG sites were studied for each particular CpG island. The primers for pyrosequencing and PCR conditions are the same as previously reported [6]. Bisulfite-treated DNA (1 μl) was amplified in 50 μl of reaction mixture, containing primers and 0.2 U of Taq polymerase (New England Biolabs, Ipswich, MA, USA). For the amplification of HIN-1, we used a universal primer approach [6]. The PCR product was purified and methylation was quantitated using the PSQ HS 96A pyrosequencing system and Pyro gold reagents (Biotage). Methylation data are presented as the average percentage of methylation in all observed CpG sites. To set the controls for pyrosequencing, we used cancer cell lines and normal cells that were consistently positive or negative with stable levels of methylation. In this study, each PCR assay included a positive control (the MDA-MB-231 breast cancer cell line, which is highly methylated in most genes) and a negative control (normal breast epithelial cells, HMEC231, which are unmethylated in all genes).

### Statistical methods and analysis

Subject and tumor characteristics were summarized in the 193 subjects with methylation data using descriptive statistics. The methylation values (expressed as the average percentage methylation of all CpG sites for a particular gene) ranged from 0 to 98% and were highly skewed. Differences in methylation levels as a function of tumor characteristics were analyzed with Wilcoxon rank sum tests, and these results were reported with adaptive Hochberg *p*-values to adjust for multiple comparisons. In addition, the median methylation level of each gene was reported for each tumor characteristic group.

For survival analysis, the gene values were divided at the median with the lower half as the reference group. Time to first recurrence (TTFR) was calculated from the diagnostic biopsy date to first recurrence. Subjects without an event were censored at the time of death (other causes) or last follow-up. Overall survival (OS) was calculated from the diagnostic biopsy date to death, censoring subjects who were alive at the time of last follow-up. For each gene, the effect of methylation on time to first recurrence and on overall survival was examined using Kaplan-Meier curves and differences in survival were evaluated with the log-rank test. Where the log-rank test was significant at α = 0.05, Cox regression was used to model the relationship, and hazards ratios and 95% confidence intervals were calculated for the univariable and multivariable models. The proportional hazards assumption was checked with graphical and time-varying covariate modeling methods. Small sample sizes precluded investigation of subgroups. All analyses were performed using SAS 9.2 (SAS, Cary, NC) and R software, version 2.10.1.

## Results

### Characteristics of the patients and breast cancers

Tumors from 193 patients with early stage breast cancer who received no adjuvant systemic therapy were used for analysis. The patients’ clinical characteristics were summarized in Table 1. Most of the subjects were Caucasian (74.86%) and post-menopausal (84.44%). Of the 193 patients, 161 cases (83.42%) were initially diagnosed as Infiltrating Ductal Carcinomas, the majority were ER + (141, 73.06%) and aneuploid (113, 58.55%) and had low-medium S-phase (102, 53.13%). Eighty-five cases (47.49%) were PR+. The median follow-up time was 152 months (range, 0–266 months).

**Table 1:** Characteristics of subjects in the dataset (N = 193)

	**Frequency (%)**	**Median**	**(Min, Max)**
Race			
White	131 (74.86%)		
Non-White	44 (25.14%)		
Menstrual Status			
Pre/Peri	28 (15.56%)		
Post	154 (84.44%)		
Histopathology			
IDC	161 (83.42%)		
ILC	13 (6.74%)		
Medullary	5 (2.59%)		
Mucinous/Colloid	6 (3.11%)		
Tubular	2 (1.04%)		
Infiltrating Papillary	2 (1.04%)		
Adenocarcinoma	2 (1.04%)		
Carcinoma, NOS	2 (1.04%)		
Tumor Size			
≤ 2 cm	97 (50.26%)		
>2 cm and ≤ 5 cm	96 (49.74%)		
Stage			
Stage I	97 (50.26%)		
Stage IIA	96 (49.74%)		
ER			
ER+	141 (73.06%)		
ER-	52 (26.94%)		
PR			
PR+	85 (47.49%)		
PR-	94 (52.51%)		
Ploidy			
Diploid	80 (41.45%)		
Aneuploid	113 (58.55%)		
S-Phase			
Low-Medium (≤10%)	102 (53.13%)		
High (> 10%)	90 (46.88%)		
RIL		18.1%	(0, 91.2)
HIN-1		13.6%	(0, 98.4)
RASSF1A		16.1%	(0, 97.1)
CDH13		8.6%	(0, 77.1)
Age		62 years	(30, 88)
Median follow-up time*		152 months	

*Determined by scoring deaths as censored events and calculating follow-up time using Cox regression.

### Association of methylation with clinical characteristics

The median values for methylation of the four genes ranged from 8.6% to 18.1% (Table 1). White subjects and non-White subjects had similar methylation levels for the genes and there was no statistically-significant difference in the methylation levels of the genes by race group (data not shown). In addition, we found that methylation values appear to differ by different tumor characteristics. Higher median methylation values were seen for all four genes with larger tumor-size (T2, 2–5 cm) than with smaller tumor-size, (T1,< 2 cm) and with aneuploid compared to the diploid cancers (data not shown).

Associations between the patients’ clinical characteristics and methylation of the four genes were evaluated using Wilcoxon rank sum test with adaptive Hochberg p-values to adjust for multiple comparisons (Table 2). ER + tumors had higher median methylation levels of HIN-1 (*p =* 0.002), and RASSF1A (*p* < 0.001), and PR + tumors had higher levels of HIN-1 (*p* = 0.002) and RASSF1A (*p* = 0.019). Lower levels of RIL (*p =* 0.012) were also found in PR + tumors. Smaller tumors (≤ 2 cm) had lower methylation levels of all four genes, and methylation of RIL and CDH13 (both *p* = 0.0020) differed significantly by tumor size group. Lower methylation levels of RIL were found in tumors with low S-phase (*p* = 0.036) and in diploid tumors (*p* < 0.001).

**Table 2:** Wilcoxon rank sum tests with p-values adjusted for multiple comparisons for gene methylation levels and tumor characteristics

**Tumor Characteristic Group**	**Gene**	**Median Methylation Levelof the Gene**		**Adaptive Hochberg *p*-value for the Wilcoxon ranked sum tests**
ER		ER+	ER-	
	RIL	16.75%	21.90%	0.58
	HIN-1	17.00%	7.90%	**0.002**
	RASSF1A	23.10%	8.90%	**<0.001**
	CDH13	8.60%	8.35%	0.58
PR		PR+	PR-	
	RIL	14.70%	24.65%	**0.012**
	HIN-1	19.50%	8.60%	**0.002**
	RASSF1A	23.10%	13.4%	**0.019**
	CDH13	8.60%	8.70%	0.14
Tumor Size		≤ 2 cm	>2 cm	
	RIL	15.45%	21.30%	**0.002**
	HIN-1	12.10%	16.30%	0.45
	RASSF1A	13.60%	21.40%	0.09
	CDH13	7.30%	9.65%	**0.002**
S-phase		≤ 10%	> 10%	
	RIL	14.80%	24.55%	**0.036**
	HIN-1	16.10%	12.65%	0.39
	RASSF1A	18.50%	15.30%	0.17
	CDH13	7.05%	9.30%	0.31
Ploidy		Diploid	Aneuploid	
	RIL	14.80%	23.70%	**< 0.001**
	HIN-1	12.45%	16.20%	0.30
	RASSF1A	14.90%	17.90%	0.93
	CDH13	7.10%	8.80%	0.07

### Association between methylation of genes and patient survival

**Figure 1:**
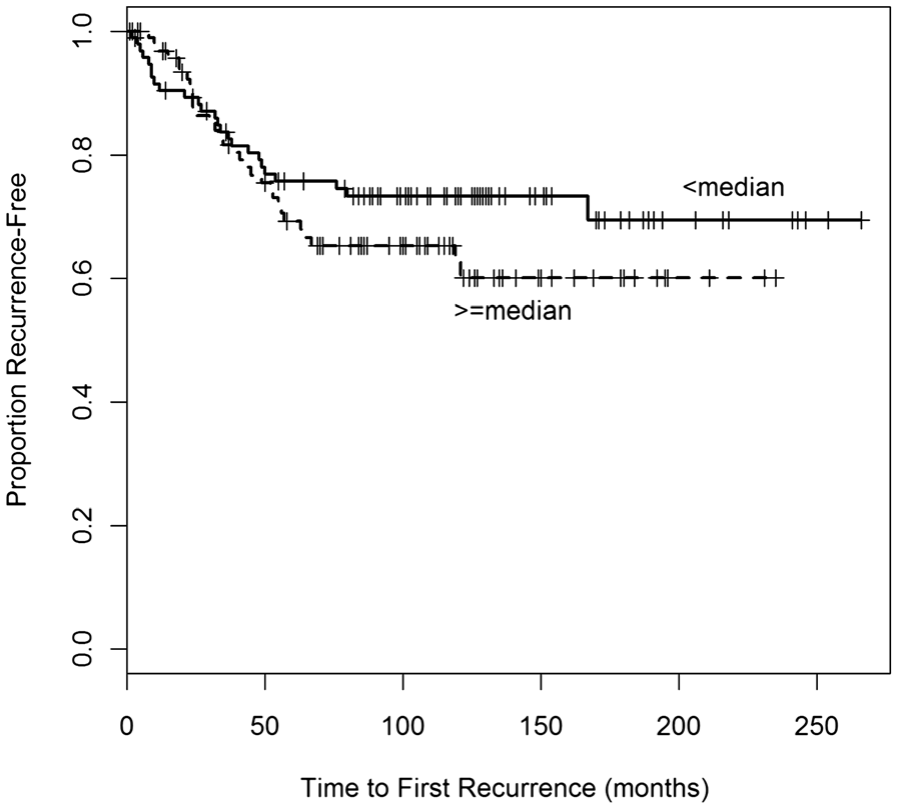
Time to First Recurrence by RASSF1A methylation status.

**Figure 2:**
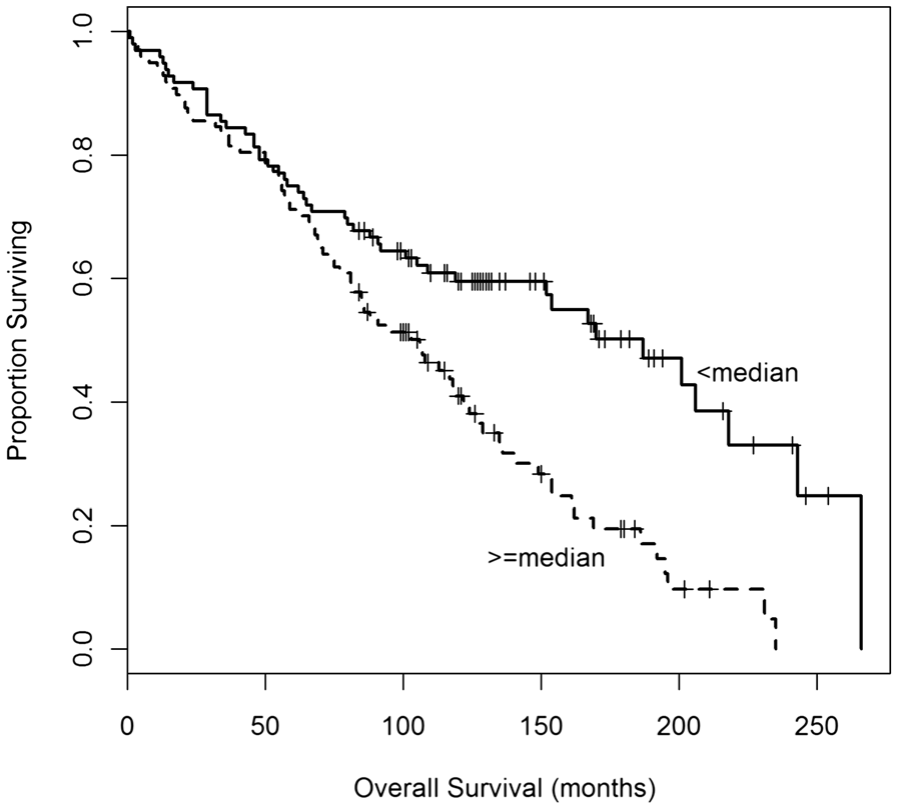
Overall Survival by RASSF1A methylation status.

As the median follow-up time for the dataset was 152 months, we were interested in seeing if methylation of the four tumor suppressor genes was associated with patient survival or time to first recurrence. We found that higher methylation of RASSF1A was associated with worse time to first recurrence {HR = 1.93, 95% CI (1.02, 3.67), *p* = 0.045} and worse overall survival {HR = 1.74, 95% CI (1.11, 2.74), *p* = 0.016} in multivariable models that included age, tumor size, S-phase, ER, and PR (Table 3, Figures 1 and 2). In a breast cancer-specific multivariable model, RASSF1A was not associated with worse breast-cancer specific survival {HR = 0.86, 95% CI (0.36, 2.06); *p* = 0.73}. Substantial violations of proportional hazards were not found in these comparisons, and there is no expectation that the conclusions were affected. Collectively, these results suggest that overall survival may have been associated with other factors that were potentially confounding factors, including lack of adjuvant therapy and the time period in which these cases were collected.

**Table 3:** Multivariable Survival Analysis Results by median methylation level (< median is the reference group)

**Gene**	**Outcome**	**HR (95% CI)**	*p*-value*
RIL	TTFR	1.53 (0.85, 2.77)	0.16
	OS	1.12 (0.76, 1.67)	0.57
HIN-1	TTFR	1.49 (0.83, 2.68)	0.19
	OS	1.21 (0.82, 1.80)	0.34
RASSF1A	TTFR	**1.93 (1.02, 3.67)**	**0.045**
	OS	**1.74 (1.11, 2.74)**	**0.016**
CDH13	TTFR	0.75 (0.42, 1.33)	0.32
	OS	1.15 (0.77, 1.70)	0.50

The models include age (continuous), tumor size (continuous), S-phase, ER (continuous) and PR (continuous).

*P-value from Cox regression model.

## Discussion

It is evident that methylation plays an important role in breast cancer development and progression. Hypomethylation of DNA may lead to chromosomal instability, while aberrant promoter CpG island hypermethylation is associated with transcriptional silencing. Tumor suppressor genes, such as RASSF1A, HIN-1, are the key targets of hypermethylation in breast cancer and therefore may lead to malignancy by deregulation of cell and growth cycles. Although there have been numerous studies of methylation in breast tumors associations between the methylation of tumor suppressor genes with clinical characteristics and outcome, a substantial limitation of these different studies is that the data are highly variable, possibly due to differences in laboratory and statistical methods, sample size and clinical status of the subjects.

Our previous report showed that methylation of 4 tumor suppressor genes (RASSF1A, HIN-1, RIL and CDH13) correlate with hormone receptor status [6]. The purpose of the current study is to replicate our original study with a larger, independent sample. In this study, we found that ER and PR were associated with RASSF1A and HIN-1; PR was also associated with RIL. We have also found that RIL methylation levels significantly differed by tumor size, S-phase and ploidy groups. CDH13 methylation levels significantly differed by tumor size group. These data are consistent with our previous findings that RASSF1A/HIN-1 were associated with hormone status; in addition, we have shown that RIL and CDH13 were associated with tumor characteristics.

This project also gave us the opportunity to examine the association between promoter methylation of the four tumor suppressor genes and prognosis. We found that hyper-methylated RASSF1A was associated with worse overall survival and worse time to first recurrence. The results from the survival analyses suggest that overall survival may have been associated with other factors, including lack of adjuvant therapy and the time period in which these cases were collected.

RASSF1A is a well studied tumor suppressor gene, which plays important role in many cell functions: apoptosis, cell cycle arrest, microtubule stabilization and metaphase arrest [21,22]. Re-expression of RASSF1A in tumor cell lines decreases *in vitro* colony formation and *in vivo* tumorigenicity [21]. DNA methylation of RASSF1A would be expected to lead to loss of function and an increase in spontaneous or induced tumor formation. Our data combined with other groups’ data suggest that RASSF1A methylation could be a potential molecular biomarker, and further replication analyses in larger cohorts and different populations are warranted. Although the results from this study indicate that the methylation of RASSF1A may be associated with overall survival, it is possible that these findings may be affected by unmeasured potentially confounding factors. It is also feasible that these results are due to chance, as different methylation levels were used to define hyper-methylation for each gene and the only positive results were found for RASSF1A.

## Conclusions

Tumors from 193 patients with early stage breast cancer were used to analyze promoter methylation of the RIL, HIN-1, RASSF1A and CDH13 tumor suppressor genes for association with known predictive and prognostic factors and for impact on time to first recurrence and overall survival. Methylation of HIN-1, RASSF1A, RIL and CDH13 were associated with a number of different clinical characteristics, but only hyper-methylated RASSF1A was associated with worse overall survival and worse time to first recurrence. These results and those of other groups suggest that further studies are reasonable to determine if RASSF1A methylation could be a potential prognostic biomarker.

## Abbreviations

ER: estrogen receptors; PR: progesterone receptors; HR: hormone receptors; TTFR: time to first recurrence; OS: overall survival.

## Competing interests

These authors report no financial or intellectual conflicts of interest regarding this study.

## Authors’ contributions

JX and WF prepared DNA, carried out the methylation assays. JX, PBS and CC prepared the manuscript. PBS performed the statistical analysis. CC provided tissue samples, clinical data. RCB and JJI conceived the study and helped to prepare the manuscript. SGH and YY conceived the study and gave final approval of the manuscript. All authors read and approved the final manuscript.

## Pre-publication history

The pre-publication history for this paper can be accessed here:

http://www.biomedcentral.com/1471-2407/12/243/prepub
